# Rectal Foreign Body: A Case Report

**DOI:** 10.31729/jnma.7905

**Published:** 2022-12-31

**Authors:** Anamika Nepal, Shailesh Maharjan, Anup Chalise, Ashish Prasad Rajbhandari

**Affiliations:** 1 Shankarapur Hospital Pvt. Ltd, Gokarneshwor, Kathmandu, Nepal; 2 Nepal Medical College and Teaching Hospital, Jorpati, Kathmandu, Nepal

**Keywords:** *case reports*, *foreign bodies*, *laparotomy*, *rectum*, *sigmoidoscopy*

## Abstract

The rectal foreign body is a rare presentation, often related to sexual gratification, sexual assault, or the result of ingestion and rarely accidental, and with rising incidence. We present a case of a 47-year-old heterosexual male with an alleged history of accidental insertion of a foreign body through the anus three days prior without peritonitis or obstipation. After investigations, the patient underwent a failed sigmoidoscopic removal followed by exploratory laparotomy, foreign body removal, and an uneventful post-operative period. It should be noted that early diagnosis and timely intervention are important to prevent complications in rectal foreign bodies. Assessment of the shape, size, nature, and location of the object through appropriate imaging is necessary. Exploratory laparotomy is inevitable in cases of failed manual extraction techniques and complicated cases.

## INTRODUCTION

Rectal foreign body is a rare presentation, often related to sexual gratification, sexual assault or the result of ingestion and rarely accidental, and with rising incidence.^1^ They are relatively common in the urban population and mostly seen in males of the 3^rd^ and 4^th^ decades.^2^ Earliest case reports of rectal foreign body date back to the 16^th^ century.^3^ Management of a foreign body in the rectum is often challenging for a surgeon due to the variation in time of insertion, associated injuries, and type and location of an object.^4^ We present a case of a rectal foreign body, the nature and shape of which made the identification and removal even more challenging.

## CASE REPORT

A 47-year-old married male (heterosexual) presented to the emergency department with an alleged history of accidental insertion of a water glass through the anus three days prior. He denied purposeful insertion initially, but later on admitted to using the glass for self-gratification purposes when he was intoxicated. He had not passed stool for two days but was passing flatus, and there was no abdominal distension. It was associated with lower abdominal and rectal pain but no per-rectal bleeding. He had tried to remove the glass himself but had been unsuccessful. There were no comorbidities, and the patient did not have any history of psychiatric illnesses. The patient's mood, behaviour patterns, and insight were normal at the time of examination.

On physical examination, the abdomen was soft and non-tender, the foreign body was not palpable, and there were no signs of peritonitis. On digital rectal examination (DRE), there was no anal injury or bleeding, and the anal tone was intact, lower margin of the glass could be felt in the upper rectum.

**Figure 1:**
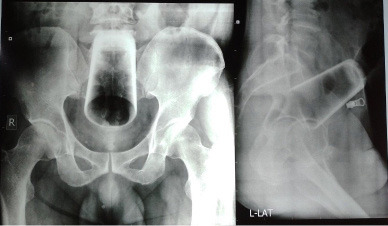
X-ray abdomen erect-AP and lateral.

The patient was admitted and investigated. The preoperative investigations were within normal limits. An erect abdominal X-ray showed a foreign body resembling a water glass in an inverted position in the upper rectum and sigmoid but no pneumoperitoneum (Figure 1).

Patient and patient party were counselled about various modalities of treatment and surgery. The appearance of the glass on imaging showed that attempting sigmoidoscopy would be futile due to the presence of features of obstruction, the size of the glass, the direction of insertion, and the likelihood of breaking it on removal. So, the patient was kept in a lithotomy position and manual removal of glass was attempted via anal opening, but the procedure was averted as the glass could not be grasped for removal, and as there was a significantly high risk of glass breakage which could, in turn, lead to injury to the bowel or to the intact anal sphincter complex.

**Figure 2:**
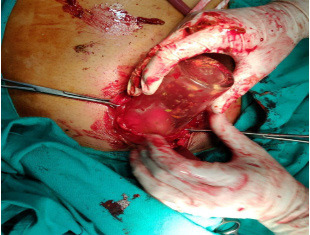
Sigmoid enterotomy to remove the glass.

**Figure 3:**
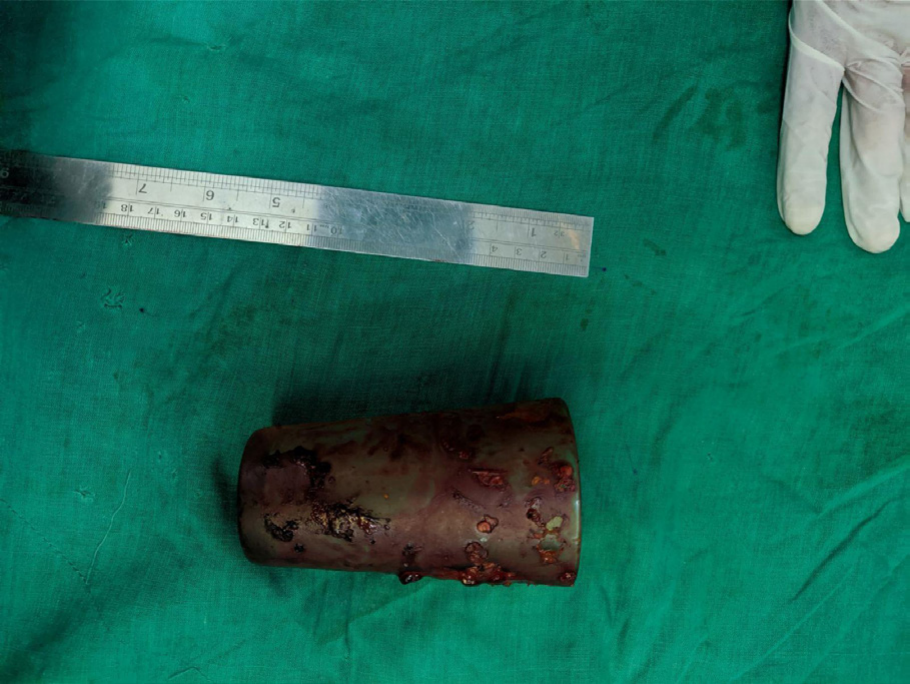
Post-operative specimen of the removed glass.

Exploratory laparotomy was performed through a lower midline incision. No evidence of bowel perforation was noted. The water glass could be felt in the sigmoid. Milking was attempted to deliver the glass through the anus, but this was unsuccessful as the glass was high up, inverted and tightly wedged. Hence sigmoid enterotomy was done and the glass was extracted (Figure 2 and Figure 3).

Primary repair of sigmoid enterotomy was done and a pelvic drain was kept. The post-operative period was uneventful. On the third postoperative day, the patient passed flatus and a liquid diet was started. The patient had passed stool passed by the fifth postoperative day. A drain was removed on the sixth postoperative day and the patient was discharged on the seventh postoperative day. On regular follow-up, after two months of the surgery he is well, and the anal tone is still intact.

## DISCUSSION

There are multiple case reports on rectal foreign bodies. Insertions of a wide variety of foreign bodies have been reported mostly plastic or glass bottles, soda or beer bottles, deodorant containers, wooden or rubber objects, and household objects.^7^ Commonly reported objects are betel nuts, bones, batteries, etc. in involuntary ingestion while beverage bottles and candles are more commonly associated with cases of sexual gratification. Iron rods, glass bottles, and wooden handles are more common among victims of sexual assault.^2^ The most common reason for a foreign body in the rectum was found to be purposefully inserted foreign body for sexual gratification in unnatural sexual behaviour.^1^ Most of the patients are usually intoxicated during the time of insertion of a foreign body.^6^ Other than sexual behaviour, a foreign body in the rectum is generally found in children, the elderly, and in patients with psychiatric illnesses.^8^ In these cases ingested objects are mostly erasers, bottle caps, and coins. Rectal foreign bodies are also common among drug traffickers known as body packing.^9^ All these objects may cause severe injury to the rectum, so they should be treated as hazardous. Our patient inserted a glass cup for the purpose of sexual gratification and denied this possibility early on, but later admitted to it.

Sub-acute intestinal obstruction is the most common presentation. The patient usually presents with abdominal pain, per-rectal bleeding, and constipation and often presents after multiple attempts of selfremoval. Presentation is usually always delayed because of embarrassment.^5^ In case of perforation they present with fever, vomiting, and severe abdominal pain, and these patients may have associated signs of sepsis. In such complicated cases (resuscitation) parenteral hydration and broad-spectrum antibiotics are indicated with urgent exploratory laparotomy.^5^ Our patient presented with signs of obstruction as the glass cup was too large to pass down through the anus, and since the mouth of the cup was wider, it lodged in the area of the anal sphincter.

Per rectal examination is vital in the diagnosis of the rectal foreign body but recommended only after proper imaging of the abdomen so that presence of sharp objects is excluded to prevent accidental injuries.^8^ X-ray abdomen and pelvis help in localizing the foreign body while computed tomography (CT) scans are sometimes necessary to rule out complications (intestinal perforation).^7^ Due to delayed presentation wide variety of rectal foreign bodies causes severe injury and damage ranging from mucosal injury to free intestinal perforation which results from the difficulty in diagnosis and management and leads to sepsis and death.^10^ These patients require urgent interventions to relieve symptoms and prevent complications. It is also essential to exclude the associated injuries and complications. Although our patient had not developed sepsis, any further delay would probably have resulted in a different outcome.

There is a wide variety of techniques used for the extraction of foreign bodies. Choosing the most appropriate method of extraction is often difficult.^5^ About 35-40% of those with associated injuries and complications necessitate urgent surgical removal. Its management depends upon size, shape, and location.^2^ About 60-75% of the rectal foreign bodies can be removed via the transanal approach.^5^ If a foreign body is present within 10 cm of the anal verge and no signs of peritonitis are present then this approach may be helpful. Similarly, foreign bodies may be removed with the aid of sigmoidoscopes or colonoscopes.

Laparotomy is indicated when transanal or endoscopic removal fails, or if complications such as bowel perforation occur.^11^ Objects presenting proximal to the rectum, if not extracted within 24 hours, laparotomy is considered the primary management method.^11^ Like in our case, the transanal approach failed and we converted to a laparotomy.

Due to delayed presentation, rectal foreign bodies may migrate to the neighbouring organ.^12^ It may cause obstruction in the cecum, appendix, ileocecal valve, and anus.^4^ Absorption of degraded material may lead to poisoning. The most dreadful complication is perforation of the bowel leading to sepsis and death. In the present case laparotomy and sigmoid enterotomy were done and timely removal of the foreign body led to a good outcome.

Early diagnosis and timely intervention are important to prevent complications in rectal foreign bodies. Assessment of the shape, size, nature, and location of the object through appropriate imaging. Exploratory laparotomy is inevitable in cases of failed manual extraction techniques and complicated cases.
